# Using identification method to modelling short term luminous flux depreciation of LED luminaire to reducing electricity consumption

**DOI:** 10.1038/s41598-023-27925-5

**Published:** 2023-01-12

**Authors:** Roman Sikora, Przemysław Markiewicz, Ewa Korzeniewska

**Affiliations:** 1 Institute of Electrical Power Engineering, Lodz University of Technology, Lodz, Poland; 2 Institute of Electrical Engineering Systems, Lodz University of Technology, Lodz, Poland

**Keywords:** Electrical and electronic engineering, Computational science

## Abstract

Reducing electricity consumption is currently one of the most significant global issues. Luminaires and light sources are characterised by relatively low rated power values. However, due to their high number, they account for a noticeable share of the total volume of electricity consumption. When the LED lamp/luminaire is switched-on, it emits a higher luminous flux and receives more power from the mains supply than when the thermal conditions have stabilized. This phenomenon is called short-term luminous flux depreciation. The lighting design process on photometric data obtained for steady-state operating conditions is based, on once the luminous flux has stabilized. Therefore, it is possible to design the control algorithm of the LED luminaire in such a way as to reduce this phenomenon, which will result in measurable savings of electrical energy. The paper proposes the use of a method to identify the short-term luminous flux depreciation of LED luminaires. The model was then used to simulate the operation of a control algorithm limiting the phenomenon of short-term luminous flux depreciation.

## Introduction

At present, about 8% of the electricity generated worldwide is estimated to be consumed by lighting^1^. Improving the energy efficiency of lighting installations is relatively simple and usually does not require large investments. The simplest way to improve the energy efficiency of lighting installations is to replace light sources or luminaires with energy-efficient ones by equipping them with a dimming function^2^. Light sources and luminaires made in LED technology are considered to be the best in terms of both energy efficiency and functionality^3–13^. They have both advantages and disadvantages. The advantages of LED light sources are, among others, lower electricity consumption, high luminous efficacy, durability, low sensitivity to ambient temperature, and above all, simple regulation of the luminous flux. The disadvantages are the generation of harmonic currents to the mains, the emission of blue light, and the need for a cooling system (heat sink) which increases the weight of the luminaire^3–13^. The proper selection of the heat sink, and its thermal resistance, ensure appropriate thermal operating conditions for LEDs. In the case of LEDs, there is a relationship between the luminous flux and the junction temperature of the LED^14,15^. When the LED lamp/luminaire is switched-on, it emits a higher luminous flux than when the thermal conditions have stabilized. As time passes, the thermal conditions of the luminaire stabilize. As time passes, the thermal conditions of the luminaire stabilize. The luminous flux decreases until it reaches the steady-state (rated) value. This phenomenon is called short-term luminous flux depreciation. For LED luminaires with large power ratings in the order of hundreds of watts, it may take several hours for the thermal conditions and thus the luminous flux to stabilize. Therefore, the standard^16^ recommends that the measurements of photometric parameters be carried out in steady-state, and for such luminaires, the recommended lighting time is up to 2 h. During the stabilization of thermal conditions, the flux decreases from a few to several percent which is confirmed by the measurement results presented in the article and literature^12–15^.

During the designing of lighting, photometric data (light distribution curves) obtained for stabilised operating conditions are used. Considering that after switching on the LED light source the luminous flux is higher than under steady-state conditions view point of ensuring the best possible lighting performance, this is a positive phenomenon. However, considering energy efficiency, this is unfortunately an undesirable phenomenon. A higher luminous flux corresponds to a higher electricity consumption by the luminaire. Increasing the power (electricity consumed by the luminaire) causes a deterioration in the energy efficiency of the device as well as the entire lighting installation. The lighting project, as previously mentioned, is developed for steady-state conditions, so reducing the luminous flux to its rated value will not cause a deterioration in lighting performance. In this way, energy efficiency can be improved and electricity consumption decreased. Using the possibilities of luminous flux (power) regulation of LED light sources^6–9,17^, it is possible to develop such a control algorithm to reduce the luminous flux value to the nominal value during the stabilisation phase of thermal conditions. Assuming practically linear dependence of active power as a function of the luminous flux of LED luminaire, a decrease in the luminous flux value will result in a decrease of the active power consumed. In Ref.^18^, new LED lamp designs using cooling systems based on heat pipes and radiators of spherical shape and in the form of rings were described. Experimental results described in Ref.^19^ confirmed the possibility of reducing the decrease in luminous flux when stabilizing the thermal conditions of LED lamps of the above-mentioned novel designs.

There are many types and designs of light sources and LED luminaires available on the market today. Developing a single universal mathematical model that can be used to build a control system is difficult. The developed model may not simulate the operation of various physical objects with the required accuracy. In addition, the determination of a dedicated model for a given luminaire or light source is labor-intensive and expensive. Nevertheless, it is the best method. Consideration should therefore be given to the method of developing such a model itself. Several LED light sources and luminaire models have been described in the literature^14,15,17,20^, but none of them can be called fully universal. Some of them are difficult to implement in a real control system. Firstly, there must be a method of determining the mathematical model, using which a model of practically any luminaire can be developed simply and quickly. The obtained mathematical model should have the possibility of simple implementation in both the IT and hardware layers. According to the authors, such a method is the identification of dynamic objects method^21–40^.

In lighting, control systems based on both conventional and intelligent controllers are used to reduce electricity consumption and ensure optimal lighting parameters in the visual task area^41–56^. These control methods are used in indoor lighting^39–50^ as well as road lighting^53–56^. Conventional control systems use PI or PID controllers. Besides many undoubted advantages such as ease of implementation, they also have disadvantages such as possessing constant parameters, large time delay, and poor control performance^41,42^.

Caicedo et al. in a paper^43^ described a controller developed based on a PI controller implementing dimming. The developed controller was used for control in a wireless lighting control system. Control signals were acquired from light sensors located on the ceiling and in the visual task area. Peruffo et al.^44^ described a lighting control system built on based a PI controller working with light and occupancy sensors. The control algorithm aimed to provide the required illuminance over the visual task area and to meet the user's preset illuminance level. Meugheuvel et al.^45^ also used a PI controller to develop a lighting control system. The control system takes into account the influence of daylight and the presence of users in the lit room. The use of control enabled a 10% reduction in electricity consumption compared to the linear programming method. In Ref.^46^, Boscarino and Moallem used a developed adaptive real-time MIMO (multi-input multi-output) controller and a lighting scene simulator to control indoor lighting. The controller cooperates with Radiance via a scene simulator. The described control method requires the prior development of mathematical models of the lighting system using DIALux. These models are then implemented in programming or computing environments such as MATLAB. Koroglu and Passino^47^ presented the developed illumination balancing algorithm (IBA). The algorithm regulates the light output while maintaining the required total uniformity. Wagiman and Abdullah^48^ presented developed a radial basis function neural network (RBFNN) predictive controller. The controller regulates the dimming level taking into account the influence of daylight. Gunay et al.^49^ used an approximate discrete-time Markov logistic regression (DMLR) model developed to control lighting and blinds. The model was developed using data obtained from the building management system (BMS) and weather station. Application of the developed model resulted in electricity savings of 25%. Xiong and Tzempelikos^50^ used model based control (MBC) to control a lighting system. The data needed to develop the model was obtained from sensors located outside the building and sensors located inside the building. The outdoor sensors measure the solar radiation and the indoor sensors measure the vertical and horizontal illuminance in the task area. The use of the developed control system has resulted in significant electricity savings. Dun^51^ described the developed indoor lighting control algorithm a fuzzy model reference adaptive system (FMRAS) algorithm based on Takagi–Sugeno model. The proposed algorithm is designed to control a lighting system based on LED luminaires taking into account daylight. Lighting control using a microcontroller was presented in Ref.^52^ by Chew et al. The input signals necessary for the control process were obtained from the presence and light sensors. The advantage of this solution is relatively low cost while providing high control accuracy compared to commercial control systems.

Recent years have also seen rapid development of street lighting control systems. Sanchez-Sutil and Cano-Ortega presented in their paper^53^ a control, monitoring, and energy-saving system for a street lighting system. The system is built with three basic components: Gateway for Street Lights System (GWSLS), Operating and Monitoring Device for Street Lights (OMDSL), and Illumination Level Device (ILD). Lighting levels are determined using the Artificial Bee Colony (ABC) optimization algorithm. Long Range (LoRa) protocol is used for information transmission. By using the developed control algorithm, significant power savings have been achieved. Mohandas et al.^54^ presented a developed street lighting control system built based on artificial neural networks (ANN) and fuzzy logic (FL). The data needed for the calculations is obtained from light sensors, motion sensors, etc. The developed system has been implemented in a real road lighting installation. Moreover, the paper presents the results of tests for several variants of system operation. Based on their study, the authors found that using this control system reduced electricity consumption by 13.5%. ZZhao et al.^55^ proposed an intelligent lighting control system for a highway tunnel. The system was built using Long Short-Term Memory (LSTM) neural networks. The purpose of the system is to reduce the electricity consumption of tunnel lighting while providing the required lighting conditions. The system uses inputs such as traffic volume, vehicle speed, and tunnel exit luminance. Based on system performance tests, a reduction in electricity consumption of 23.61% on sunny days and 31.40% on cloudy days was obtained. De Paz et al. in their work^56^, described an intelligent control system for public lighting. The primary objective of the control is to achieve electricity savings while providing the required lighting performance and visual comfort in the illuminated areas. They used artificial neural networks (ANN), multi-agent systems (MAS), EM algorithm, methods based on ANOVA, and a Service Oriented Approach (SOA) to develop the control system.

The process of identifying dynamic objects involves creating a model of it based on the knowledge derived from the measurement data. This process is multi-step and its theoretical basis was created by Ljung and described in Ref.^22^. Due to the continuous development of knowledge in this area, this topic has been extensively covered by the author over the last years in Ref.^23,25^ among others. A convenient tool to support the identification process in the MATLAB environment is the Identification Toolbox, which was developed by Ljung^25^. Perspectives on the development of this field of science were described by Noël and Kerschen^26^. In Schön et al.^27^ described the process of creating mathematical models using selected examples in the state space. Depending on the type of phenomenon being modeled, it can have different forms. The use of the ARMAX model is detailed in Ref.^28^, and the use of the Hammerstein model in the time domain is described in Ref.^29^. Greblicki^30^ presented the possibilities of using a nonlinear model to describe a dynamic object when the analysis of input data already at the initial stage of the identification process suggests such a solution.

The identification results are used to select the optimal controller settings for controlling a given object in a closed-loop object-controller system and discussed by many authors. Jianhong and Ramirez-Mendoza present in Ref.^31^ a new solution to replace the unknown and nonlinear feedback controller with a single approximate linear controller. In paper^32^, Zhang and Xu present a concept for identifying the equivalent inertia constant of a closed-loop power system using a test signal with a given spectrum. The frequency and active power responses measured by the phased array are then used to identify the closed-loop. According to the authors, when compared with the conventional transient signal-based method, the proposed method is simple to implement and does not degrade system security. Forssell and Ljung describe and analyze in Ref.^33^ a closed-loop identification method for fitting a model to data with arbitrary frequency weighting. This is in opposition to other methods, such as the indirect method and the two-step method, which assume linear feedback. Recently, there has been a trend to study the identification of linear systems with high-order finite impulse response (FIR) models using the regularized least-squares method. An important part of this approach is to solve the hyperparameter estimation problem, which is usually non-convex. This issue was investigated by Chen and Ljung^34^. In the paper^35^ Stefanoiu and Culita presented a PID-based control strategy for a two-tank plant, namely ASTANK2. Moreover, they provided a comparative analysis of the four regulators in terms of performance and robustness. In Ref.^36^, Bai et al. investigate the use of identifying coherent spatial–temporal structures from multivariate data, benefiting from a strong connection to nonlinear dynamical systems via the Koopman operator. In this way, it is possible, according to the authors, to identify a low-order model from limited input–output data and reconstruct the associated full-state dynamic modes using compressive sensing.

A practical implementation of these issues for controlling example objects is discussed in Ref.^37,38^. Shi et al.^37^ propose a model structure for simulating the electromechanical transients of a photovoltaic power plant and a set of circuits for studying the transient characteristics of photovoltaic power sources based on a fault simulation device. The authors state that the known models of power electronic devices are difficult to adapt to the requirements of simulation of electromechanical transients of the power system, and their parameters are difficult to obtain. In Riba et al.^38^ propose a white-box approach for parameter identification of DC-DC buck and boost converters. It is based on the discretization of the differential equations that describe their dynamic behavior. Based on the mathematical equations in discrete form and the experimental data, the parameters of the converters are identified, thus determining the values of the passive elements.

This paper presents the use of a dynamic object identification method to develop a short term luminous flux depreciation model. The models were developed for four luminaires used in road lighting using the Identification Toolbox from MATLAB. Due to the nature of the phenomenon, it was possible to use a linear model in the form of a transfer function. Then, to present the possibility of using the developed model to control LED luminaires, a model of the control system was made in the MATLAB—Simulink environment. Moreover, potential savings in electricity obtained as a result of limiting the phenomenon of short-term luminous flux depreciation were calculated.

## Method for identifying dynamic objects

The identification process involves creating a mathematical model of the object under study based on its input and output signals. The mathematical model describes the relationship between the input and output signals of the system. Models of dynamic systems are usually described by differential equations, transfer functions, and state-space equations^23,24^. Depending on the type of object and the modeled phenomenon, dynamic models can be divided into linear and non-linear. During the identification of an object (system), an appropriate model structure to fit the phenomenon must be selected. The choice of the model structure is dictated by the knowledge of physical phenomena involved in the operation of the analyzed object. The model should with assumed accuracy model the physical phenomenon and possibly have a relatively simple structure. The Black-Box method of using different mathematical structures can also be used to determine the model.

In the process of identifying a dynamic system, it is important to properly prepare the measurement data. The measured data should adequately represent the system dynamics. Appropriate performance of measurements should ensure knowing the response of the tested object to the given forcing with the assumed accuracy. Moreover, the timing of the measurement must be chosen to capture the system dynamics that are the purpose of the modeling. If the measurement time is well chosen, it allows determining the relevant time constants of the object under test. In addition, data should be measured with a sampling rate appropriate to the physical phenomenon, which is the subject of investigation.

One of the simplest, but also most commonly used in industry, e.g. for tuning PID controllers, are continuous-time process models. Continuous-time process models are low-order transfer functions and are linear models. These transfer functions describe the system dynamics using static gain, time delay, and characteristic time constants. The transfer function can take different forms, with a given number of poles and zeros, with or without an integrator, and including a time delay^24–26^.

The linear model has a relatively uncomplicated structure and in many cases its accuracy is satisfactory. A linear dynamic object in the time domain can be described by the following mathematical relation:

1 $$y\left(t\right)=G\left(s\right)\cdot x\left(t\right)+v\left(t\right).$$

For the adopted description, respectively the signal *x*(*t*) is the forcing, *y*(*t*) is the response of the object to the given forcing and the signal *v*(*t*) is the noise accompanying the operation of the tested object. This model has one input and one output (SISO). The identification process, in this case, consists in searching for the best form of the transfer function *G*(*s*) to obtain the best possible fit.

The structure adopted and the complexity of the model depends on factors such as the response of the real object, the capabilities of the tools used for identification, and the experience of the investigator. In the identification process, after selecting the model structure and comparing its response with the response of the test subject, the model structure may need to be changed. This happens when the differences in the response of the model and the real object are pronounced and it is then necessary to take a step back in the research process and use a different class of models.

Assuming a model structure in the form of a transfer function, the model is created from the following elements: time constants, a gain factor, and a delay element. The simplest form of transfer function occurs when there is no delay or inertia in the object under test. In this case, the transfer function is equal only to the gain factor *K*_*p*_ between the input and output signals (Eq. 2).

2 $$G\left(s\right)={K}_{P}.$$

In the case where there is a time constant between the input signal and the output signal, for example, due to the occurrence of thermal capacitance, a time constant element *T*_*p*_ appears in the transmittance and the transfer function is described by Eq. (3).

3 $$G\left(s\right)=\frac{{K}_{P}}{1+{T}_{P}\cdot s}.$$

In addition, if there is a delay between the input and output signals, a delay time constant *T*_*d*_ appears in the transmittance (Eq. 4).

4 $$G\left(s\right)=\frac{{K}_{P}}{1+{T}_{P}\cdot s}\cdot {e}^{-{T}_{d}\cdot s}.$$

The physics of phenomena occurring during the operation of the object under test may force the occurrence of not one time constant of the object, but two or more. The transfer function then takes the form described by Eq. (5).

5 $$G\left(s\right)=\frac{{K}_{P}}{\left(1+{T}_{P1}\cdot s\right)\cdot \left(1+{T}_{P2}\cdot s\right)}\cdot {e}^{-{T}_{d}\cdot s}.$$

If the accuracy of the model is unsatisfactory then the model structure can be further changed. As already mentioned, the selection of the appropriate form of the transfer function *G*(*s*) is made based on, first of all, the physical phenomena accompanying the operation of the object, obtaining the desired accuracy of the model while keeping its form as simple as possible. The simple structure of the model makes it easy to implement the developed model in both software and hardware layers.

**Figure 1:**
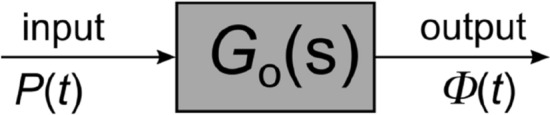
The structure of the model luminaire.

The research described in the article aims to develop a model of short-term luminous flux depreciation during thermal conditions stabilization of LED luminaire. For this purpose, measurements of LED luminaires were performed, and the results of these measurements are described in "Results of investigation". Based on the obtained results analysis, it was assumed that the input signal of the model is the active power of the luminaire and the output signal is the luminous flux. The concept of the structure of the model is shown in Fig. 1.

By analyzing the relationship between the input signal (*P*(*t*)) and the output signal ((*t*)), it was concluded that the use of a linear model—low-order transfer functions—is sufficient for mapping with the assumed accuracy. The identification results are presented in "Conclusions".

## Results of investigation

### Electrical parameters and luminous flux measurements of LED luminaires

The purpose of the research is to determine the changes in electrical parameters and luminous flux during the process of stabilizing thermal conditions of road luminaires made in LED technology. The measurement results were used to develop short-term luminous flux depreciation models of these luminaires using the dynamic object identification method^24–26^. Four road luminaires with power ratings of 32 W (luminaire labeled L1), 40 W (luminaire labeled L2), 75 W (luminaire labeled L3), and 100 W (luminaire labeled L4) were selected as test objects. Before the measurements, the luminaires were illuminated for at least 100 h according to the Ref.^16^. It was assumed that for all tested luminaires the measurement time is 1 h. Moreover, It has been assumed that for tested luminaires the thermal conditions of the luminaires will stabilize after 1 h.

Electrical parameters were measured using a FLUKE 1760 power quality analyzer. The luminaires are supplied with voltage from the LV distribution network. This type of power supply has been deliberately done so that the luminaires are supplied with the voltage they will be supplied with under real conditions. Table 1 summarises the results of selected measurements of electrical parameters and luminous flux averaged for the last minute of the measurement during the hourly measurement period. The luminous flux was measured with a 2 m diameter integrating sphere and an L-100 luxmeter.

**Table 1:** Electrical parameters and luminous flux.

Luminaire	Active power *P* (W)	Current *I* (A)	Reactive power *Q* (var)	Displacement power factor *PF*_*D*_	Distortion power factor *PF*_*DD*_	Current total harmonic distortion factor *THD*_*I*_ (%)	Luminous flux (lm)
L1	32.46	0.16	15.13	0.91	0.88	14.98	2907
L2	41.56	0.20	15.99	0.93	0.91	15.72	4735
L3	76.42	0.36	25.54	0.95	0.93	15.85	6997
L4	98.20	0.44	22.04	0.98	0.97	8.86	15,028

**Figure 2:**
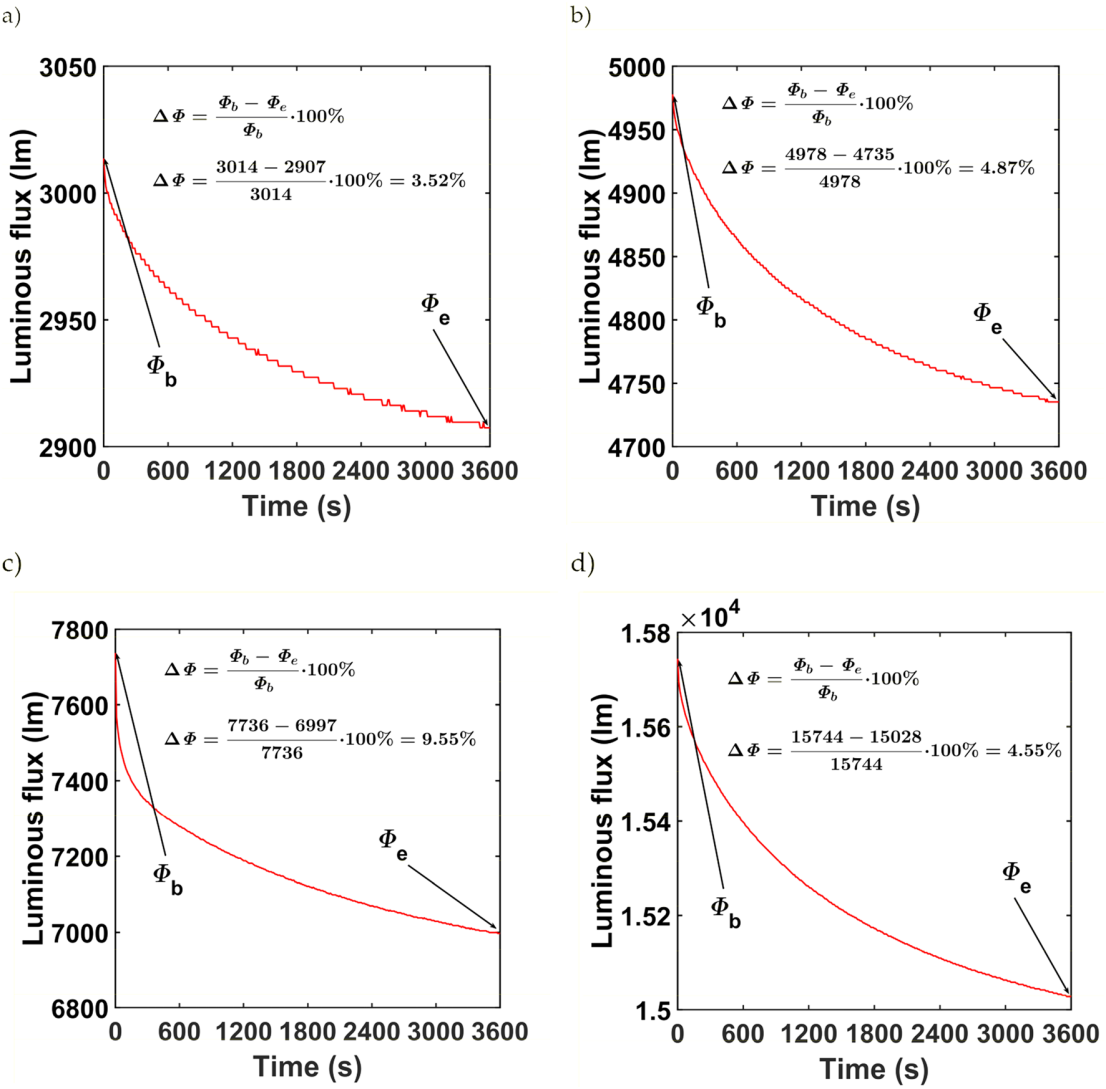
Short-term luminous flux depreciation of (**a**) L1 luminaire, (**b**) L2 luminaire, (**c**) L3 luminaire, (**d**) L4 luminaire.

**Figure 3:**
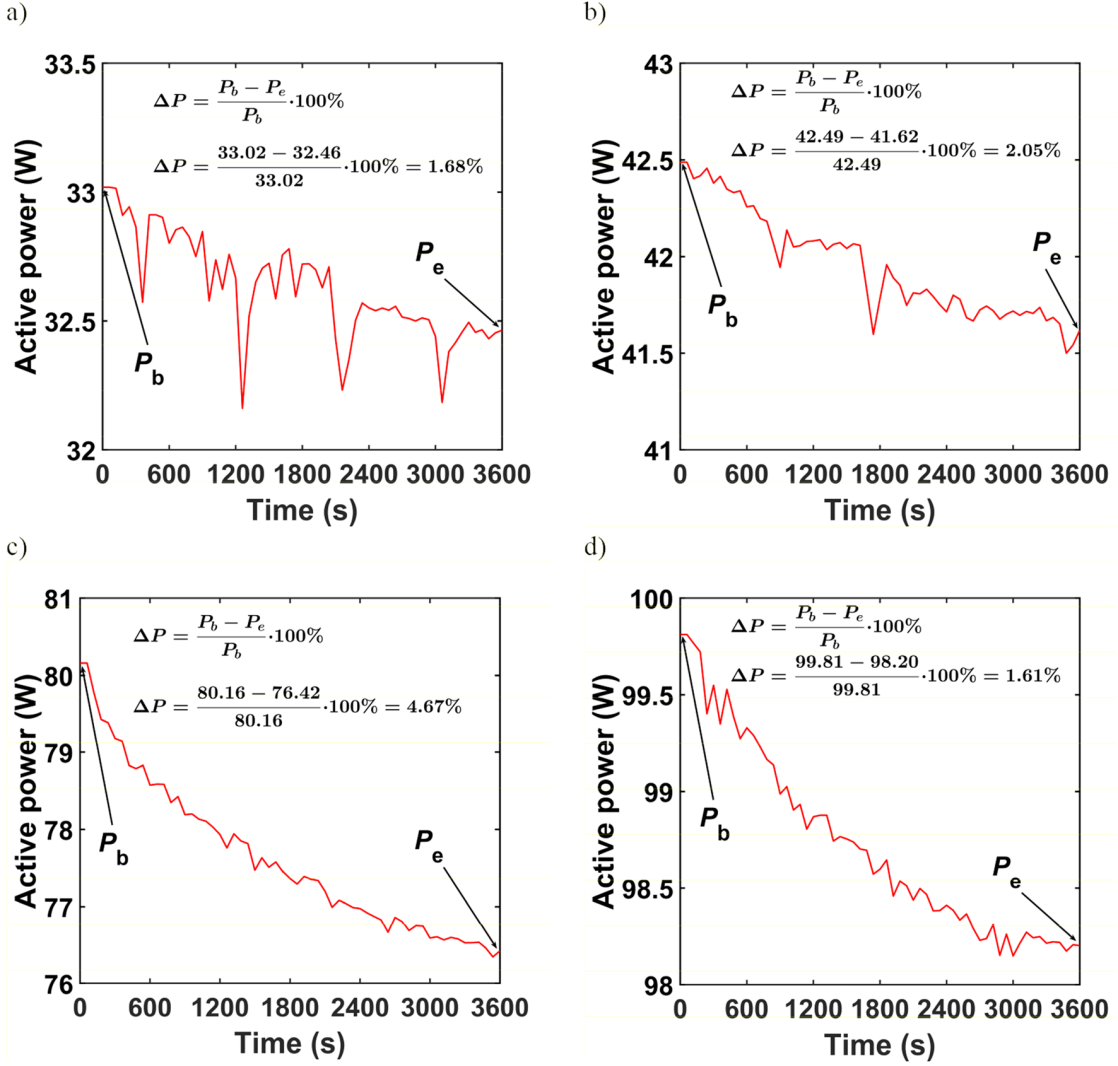
Active power during stabilisation of thermal conditions for (**a**) L1 lumianire, (**b**) L2 luminaire, (**c**) L3 luminaire, (**d**) L4 luminaire.

Short-term luminous flux depreciation due to stabilization of the thermal conditions of the tested luminaires is shown in Fig. 2. The greatest difference between the initial luminous flux _b_ and the final luminous flux _e_ occurs for luminaire L3 and is 9.55%. The smallest difference between the initial and final luminous fluxes was for luminaire L3, where it was 3.52%. By analyzing the curves of active power during stabilization of thermal conditions, it can be stated that the changes have a similar character to the changes in luminous flux. Figure 3a–d show respectively the active power curves during the stabilization of the thermal conditions of the L1, L2, L3, and L4 luminaires. For the three luminaires L1, L2, and L4, the difference between the initial value of the active power *P*_b_ and the final value *P*_e_ does not exceed 3%. For luminaire L3 the difference between *P*_b_ and *P*_e_ is 4.67%. Table 2 summarises the initial and final values of active power and luminous flux and their percentage differences.

**Table 2:** Initial and final values of active power, luminous flux and their percentage differences of the tested luminaires.

Luminaire	*P*_b_ (W)	*P*_e_ (W)	*∆P* (%)	*Φ*_e_ (W)	*Φ*_b_ (W)	*∆Φ* (%)
L1	33.02	32.46	1.68	2907	3014	3.52
L2	42.49	41.62	2.05	4735	4978	4.87
L3	80.16	76.42	4.67	6997	7736	9.55
L4	99.81	98.20	1.61	15,028	15,744	4.55

Analyzing the obtained measurement results, attention should also be focused on the values of the displacement and distortion power factors. The L1 luminaire displacement power factor is equal to 0.91. In many countries, the permissible tan value is 0.40^40^ which is equivalent to a displacement power factor value of 0.928. In this case, the use of such a luminaire could result in an increase in electricity fees due to penalties for over-contracted reactive power consumption. The *THD*_I_ values of the tested luminaires range from 8.86% for luminaire L4 to 15.85% for luminaire L3. Because the luminaires receive a distorted current (which characterizes the *THD*_I_ value) the values of the distortion power factor are smaller than those of the displacement power factor. It should also be noted that the power factor of all the tested luminaires is capacitive character.

Luminaires L1 and L2 have the lowest reactive power consumption, and luminaire L3 has the highest reactive power consumption. Despite the highest power rating, the total harmonic distortion factor *THD*_I_ of the L4 luminaire is the lowest and equals 8.86%. The *THD*_I_ values of the other three luminaires are comparable and do not exceed 16%. *THD*_I_ values of no more than 20% are acceptable, although this is not a design optimized to reduce emissions of higher harmonics. Luminaires with less than 10% *THD*_I_ (such as the L4 luminaire) can be considered a good design solution in terms of reducing the emission of higher harmonics into the mains.

### Identification of short-term luminous flux depreciation

Measurement data of selected LED luminaires used in road lighting were used to identify the phenomenon of short-term luminous flux depreciation. It was assumed that the input signal (forcing) of the model is the active power consumed by the luminaire and the output signal (response) is the luminous flux. The System Identification Toolbox, which is part of the MATLAB environment, was used to identify short-term luminous flux depreciation. Based on the analysis, a linear model of the investigated object in the form of a transfer function was selected. Identification began by assuming a simple model structure described by Eq. (2). If the assumed accuracy was not achieved, as measured by an *R*^2^ mean square error of 0.92, the model structure was modified by extending the transmittance with time constants or delays.

The luminaires selected for testing are different constructions. This is the reason that the short-term luminous flux depreciation phenomenon has a different pattern for each luminaire. Thus, it was expected to obtain other forms of mathematical models—transfer function. Table 3 summarizes the determined mathematical models of the short-term flux depreciation phenomenon. In addition, Table 3 shows the values of gain coefficients, lags, and time constants for each of the analyzed objects and the percentages indicating the accuracy of the fit.

**Table 3:** Identification models.

Luminaire	Model	Coefficients	Fit—*R*^2^
L1	$G\left(s\right)={K}_{p}\frac{1+{T}_{z}\cdot s}{1+2\cdot \zeta \cdot {T}_{w}\cdot s+{\left({T}_{w}\cdot s\right)}^{2}}\cdot {e}^{-{T}_{d}\cdot s}$	*K*_*p*_ = 89.216 *T*_*w*_ = 0.20125 = 4.3779 *T*_*d*_ = 0.00094 *T*_*z*_ = 0.013756	98.10%
L2	$G\left(s\right)={K}_{p}\frac{1+{T}_{z}\cdot s}{1+2\cdot \zeta \cdot {T}_{w}\cdot s+{\left({T}_{w}\cdot s\right)}^{2}}\cdot {e}^{-{T}_{d}\cdot s}$	*K*_*p*_ = − 57.684 *T*_*w*_ = 0.082097 = 3.8398 *T*_*d*_ = 0.01707 *T*_*z*_ = − 6.2928e−06	93.72%
L3	$G\left(s\right)=\frac{{K}_{p}}{1+{T}_{p1}\cdot s}\cdot {e}^{-{T}_{d}\cdot s}$	*K*_*p*_ = 90.516 *T*_*p1*_ = 2.0259 *T*_*d*_ = 0.02665	97.45%
L4	$G\left(s\right)=\frac{{K}_{p}}{\left(1+{T}_{p1}\cdot s\right)\cdot \left(1+{T}_{p2}\cdot s\right)}\cdot {e}^{-{T}_{d}\cdot s}$	*K*_*p*_ = − 0.019887 *T*_*p1*_ = 1.0994 *T*_*p2*_ = 0.040384 *T*_*d*_ = 0.4	97.47%

**Figure 4:**
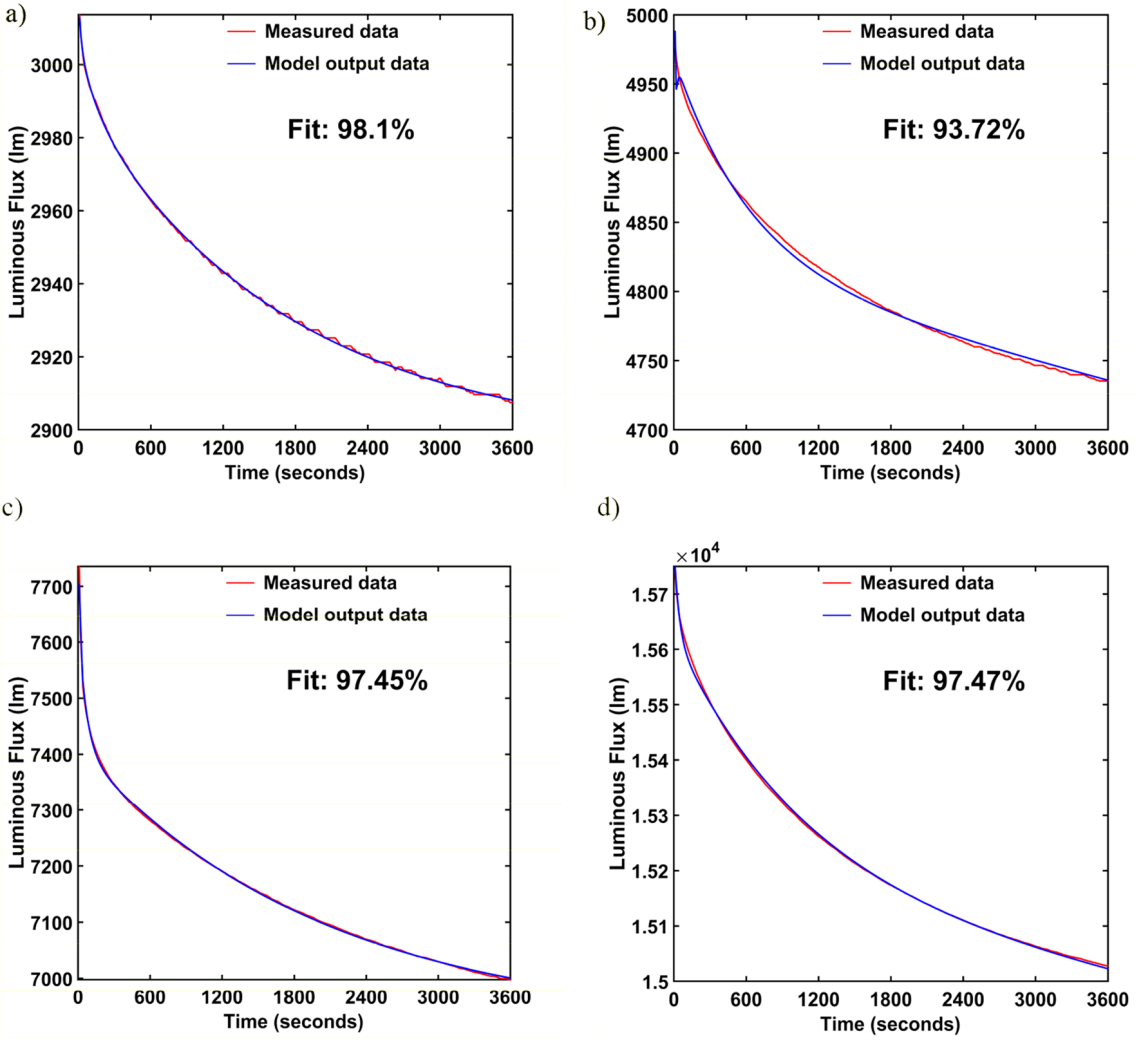
Identification of (**a**) L1 lumianire, (**b**) L2 luminaire, (**c**) L3 luminaire, (**d**) L4 luminaire.

The best-fitting accuracy was obtained for luminaire L1, where the fitting coefficient value is 98.1%. The smallest fit occurred for luminaire L2, where the fit value coefficient is equal to 93.72%. Figure 4a–d show comparisons of the time-domain responses of the test objects and their mathematical models for comparison. The obtained mathematical models will be implemented in a luminous flux control system which will be presented in "Discussion". Of course, one could continue the identification process to obtain a better fit, but this would result in a more complex mathematical model. Since the research aimed to develop a model with the simplest possible structure that could be implemented in a luminaire control algorithm, the simplest model that fulfills the assumed accuracy was adopted.

## Discussion

**Figure 5:**
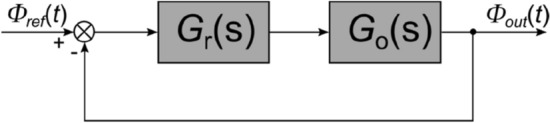
Example schematic of the control system *Φ*_*ref*_ reference luminous flux, *Φ*_*out*_ output luminous flux.

In lighting design, the value of the luminous flux measured at a steady state is used in the design calculations. This does not take into account the value of the initial luminous flux, which may be as much as about 10% greater than the rated flux specified for steady-state. Therefore, a control algorithm can be used that will reduce or eliminate short-term luminous flux depreciation. This will not negatively affect lighting conditions and will save electricity. As described in "Results of investigation", the active power value also decreases as the thermal conditions of the LED luminaire stabilize. If the control algorithm is used to stabilize the luminous flux at the time of the luminaire illumination at the nominal value, the active power received by the luminaire will also be equal to the nominal power. Using an uncomplicated control algorithm, for example, one based on a PID controller and a luminaire model obtained by using the identification method, measurable energy savings can be achieved. An additional advantage is that the proper functioning of the control system does not require signals from sensors e.g. illuminance, which is then recalculated to the value of the luminous flux. An example schematic of the control system is shown in Fig. 5.

**Figure 6:**
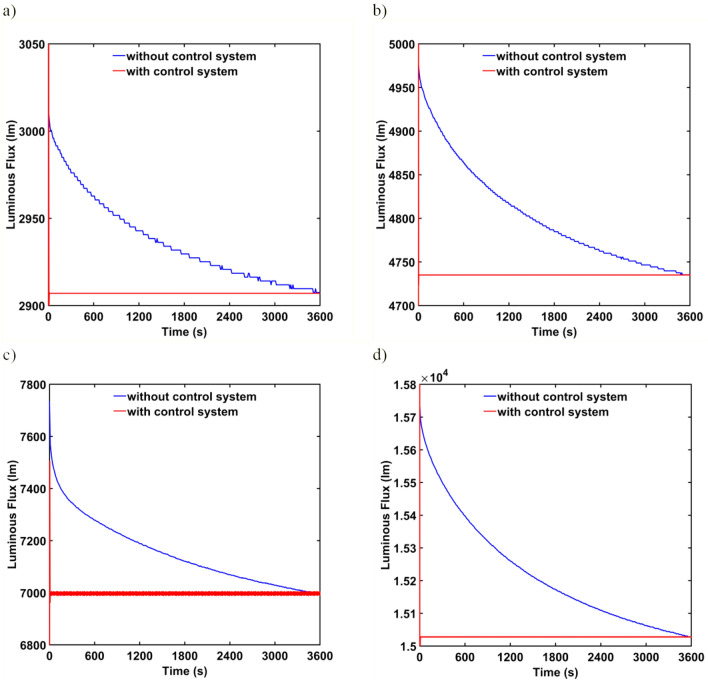
Control of (**a**) L1 lumianire, (**b**) L2 luminaire, (**c**) L3 luminaire, (**d**) L4 luminaire.

To verify the possibility of using the developed short-term luminous flux depreciation model in the LED luminaire control system, the model and simulations were performed in MATLAB / Simulink environment. The simulation results of the control system along with the comparison of operation without the control system are shown in Fig. 6 for the considered luminaires. On the basis of the simulations, it can be concluded that the model can be used in the control system of the luminaire and practically eliminates the short-term luminous flux depreciation phenomenon.

The electricity savings of *A*_*S*_ can be easily estimated as the difference in energy received from the power grid without the control system *A*_*wc*_ and with the control system that limits short-term luminous flux depreciation *A*_*c*_.

6 $${A}_{s}={A}_{wc}-{A}_{c}.$$

The energy received from the mains by the luminaire without control system can be calculated from Eq. (7).

7 $${A}_{wc}={\int }_{{t}_{b}}^{{t}_{e}}{p}_{lum}^{wc}dt.$$

Knowing the changes in active power it is possible to easily calculate the energy consumed by the analyzed luminaires using numerical integration. Analogically, the energy received by the luminaires with the implemented control system can be calculated. If the luminous flux does not change, the value of active power will not change either. Therefore, it can be assumed that the active power when the control system *P*^*c*^_*lum*_ is used is constant and equal to the fixed active power *P*_*e*_, that is, *P*^*c*^_*lum*_ = *P*_*e*_ = const.

8 $${A}_{c}={\int }_{{t}_{b}}^{{t}_{e}}{p}_{lum}^{c}dt={\int }_{{t}_{b}}^{{t}_{e}}{p}_{e}dt.$$

Knowing the time of stabilization of thermal conditions, Eq. (8), it is possible to easily calculate the value of energy received by luminaires with using the control system (Eq. 10).

9 $$\Delta t={t}_{e}-{t}_{b},$$

10 $${A}_{c}={P}_{e}\cdot \Delta t.$$

**Figure 7:**
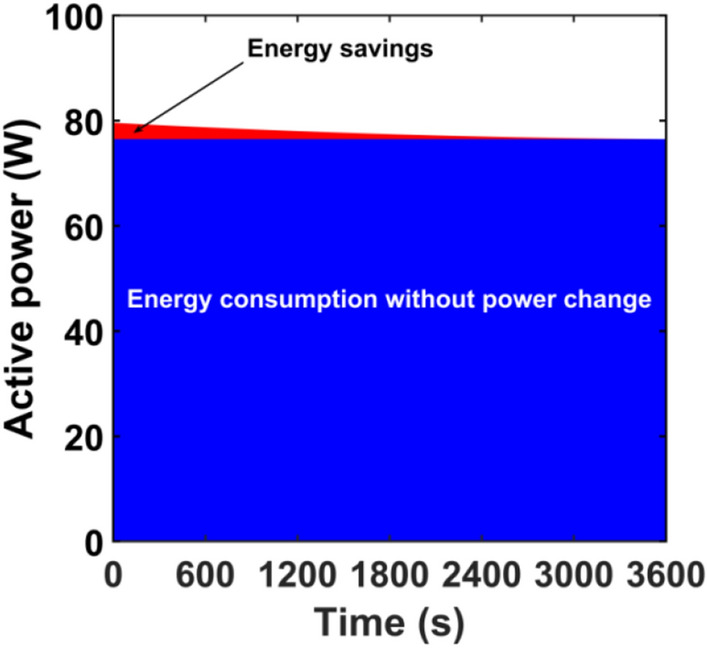
Illustration of how energy savings are calculated.

A graphical representation of the method for calculating electricity savings is illustrated in Fig. 7.

Since all calculations were performed in MATLAB, the application of the integration method required the determination of functions that approximate the changes in active power as a function of time. The approximation functions were determined using the Curve Fitting Toolbox and are summarized in Table 4. The mean squared error for the three luminaires L2, L3, and L4 did not exceed 0.986. Only for luminaire L1 the value of the mean squared error was equal to 0.955. As can be observed, with the assumed accuracy, the active power curves could be approximated by a polynomial of degree 2.

**Table 4:** Fit function of active power.

Luminaire	Fit function	Fit—*R*^*2*^
L1	*P*(*t*) = 3.123e−08*t*^2^ − 0.0002706*t* + 33.02	0.955
L2	*P*(*t*) = 5.039e−08*t*^2^ − 0.0004244*t* + 42.51	0.995
L3	*P*(*t*) = 1.884e−07*t*^2^ − 0.00154*t* + 79.56	0.991
L4	*P*(*t*) = 1.283e−07*t*^2^ − 0.0009013*t* + 99.79	0.986

**Figure 8:**
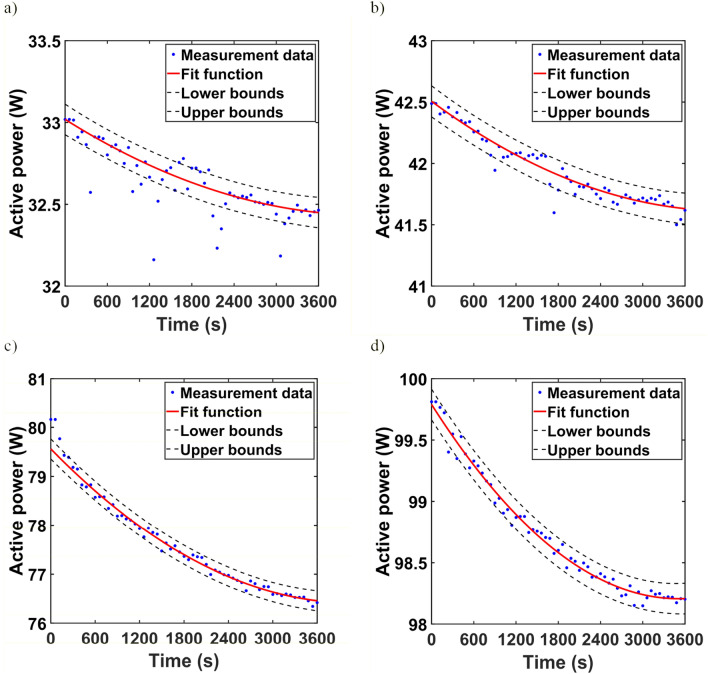
Active power of (**a**) L1 lumianire, (**b**) L2 luminaire, (**c**) L3 luminaire, (**d**) L4 luminaire.

Figure 8a–d show the active power curves obtained from the measurements and the waveforms of the approximating functions for luminaires L1, L2, L3, and L4, respectively. The 95% bounds are also marked with dashed lines.

As mentioned above, the energy received by luminaires operating without the control system was calculated using approximation functions. The energy consumed by luminaires with the control system was calculated from Eq. (10) assuming *t* = 1 h. The calculated annual energy savings obtained by using the control system are shown in Table 5 for a single luminaire.

**Table 5:** Annual electricity consumption savings per 1 luminaire.

Luminaire	Annual energy without control system per 1 luminaire *E*_*WCS*_ (kWh/year)	Annual energy with control system per 1 luminaire *E*_*C*S_ (kWh/year)	Annual energy savings per 1 luminaire *E*_S_ (kWh/year)
L1	11.92	11.84	0.08
L2	15.32	15.20	0.12
L3	28.32	27.91	0.41
L4	36.03	35.85	0.18

**Figure 9:**
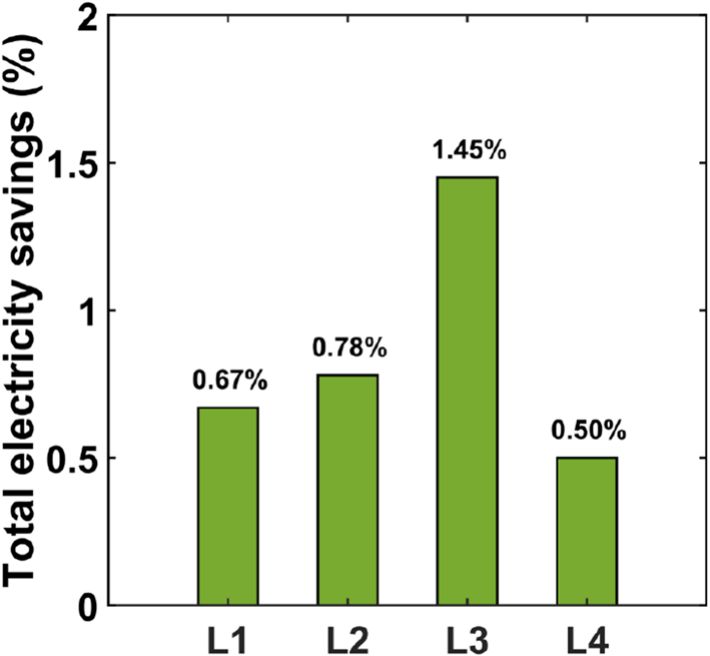
Total electricity saving.

For a single light point, the expected energy savings for the assumed annual operating cycle are between 0.08 kWh (for luminaire L1) and 0.41 kWh (for luminaire L3). For the single light point analysis, the projected savings appear to be insignificant (Table 5). Only the results of the analysis taking into account the scale effect in this case allow the right conclusions to be reached. If we assume that the series of a given luminaire type is 20,000 units and the lifetime is equal to 15 years, we can estimate the electricity savings obtained by minimizing the short-term luminous flux phenomenon. The calculations of electricity consumption of luminaires without and with the control system minimizing this phenomenon and the calculated electricity savings are summarised in Table 6. The estimated electricity savings range from 24 to 123 MWh (Fig. 9).

**Table 6:** Total electricity consumption and savings per 20,000 luminaires and 15 years performance.

Luminaire	Total energy without control system *E*^*an*^_*WCS*_ (MWh)	Total energy with control system *E*^*an*^_*C*S_ (MWh)	Total energy savings *E*^*an*^_S_ (MWh)
L1	3576	3552	24
L2	4596	4560	36
L3	8496	8373	123
L4	10,809	10,755	54

**Figure 10:**
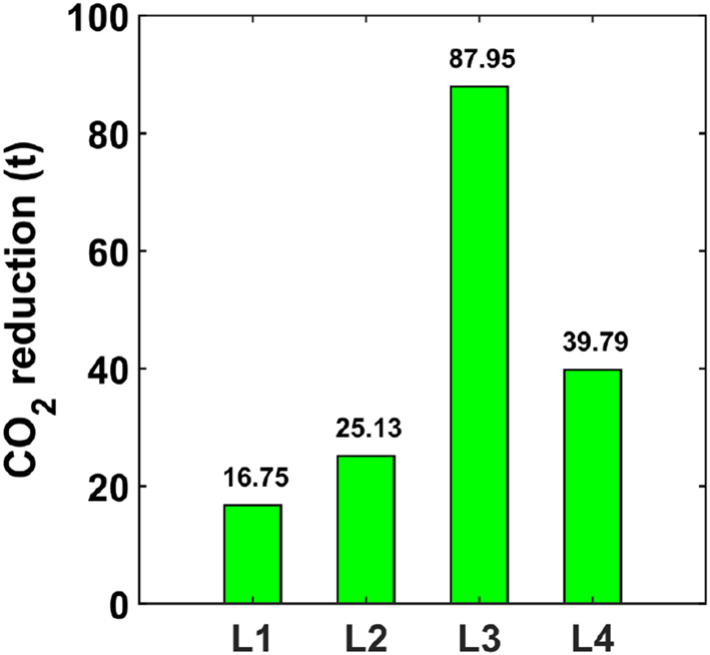
CO_2_ reduction.

If electricity is produced using fossil fuels, the savings in predictable electricity consumption results in a reduction of greenhouse gas emissions and other pollutants into the atmosphere. For the calculation of greenhouse gas and total dust emissions, the following values of emission factors were assumed for electricity end-users: CO_2_—698 (kg/MWh), SO_X_/SO_2_—0.509 (kg/MWh), NO_X_/NO_2_—0.522 (kg/MWh), CO—0.203 (kg/MWh) and total dust—0.026 (kg/MWh). Table 7 shows the estimated reductions of CO_2_, SO_x_, NO_x_, CO, and total dust for the considered luminaires and is illustrated in Fig. 9. The emission reductions were calculated using information from Ref.^57^. The largest reductions occurred for carbon dioxide. For the L1 luminaire, emissions were reduced by 16 tons, and for the L3 luminaire, emissions were reduced by 87 tons. The greatest reduction in CO_2_ and other pollutant emissions was achieved for the L3 luminaire (Fig. 10).

**Table 7:** Reduction of greenhause gases and other pollutant.

Luminaire	Carbon dioxide (CO_2_) (t)	Sulfur oxides (SO_X_/SO_2_) (kg)	Nitric oxides (NO_X_/NO_2_) (kg)	Carbon oxide (CO) (kg)	Total dust (kg)
L1	16.75	12.22	12.53	4.87	0.62
L2	25.13	18.32	18.79	7.31	0.94
L3	87.95	64.13	65.77	25.58	3.28
L4	39.79	29.01	29.75	11.57	1.48

The main objective of the proposed modification of the luminaire control algorithm to reduce the short-term luminous flux phenomenon is the possibility of improving the energy efficiency of the installation by reducing electricity consumption. The estimated savings per light point are not significant, but here the economies of scale resulting from the number of light points will play a major role. Practical implementation of the model into the control system of a luminaire is easy to perform for luminaires equipped with a microcontroller-controlled power supply, by modifying the program contained in its memory.

## Conclusions

Improving energy efficiency is now one of the most important aspects considered during equipment design. Nowadays, according to the EU Ecodesign directive^43^, every new device should be designed, operated, and disposed of in accordance with these requirements. Ecodesign requires the designer to search for all possible ways to reduce electricity consumption while maintaining the required functionality and acceptable production costs.

In the article, the application of the dynamic systems identification method to the development of mathematical models reproducing the phenomenon of short-term luminous flux depreciation of LED luminaires used in road lighting is presented. Four LED luminaires differing in construction were chosen for the study. During the research, it was assumed that the created mathematical model should have a relatively simple structure easy for hardware implementation and at the same time reproduce the phenomenon of short-term luminous flux depreciation with sufficient accuracy. The above assumptions were met by the model in the form of a transfer function. As was confirmed by the research, the transfer function can be used to model the process of short-term luminous flux depreciation with satisfactory accuracy. For the L1, L3 and L4 luminaires, the fit accuracy was 98.10%, 97.45% and 97.47%, respectively. Therefore, it can be concluded that the transfer function obtained by identification models the short-term luminous flux depreciation phenomenon with sufficient accuracy.

The determined models were used to develop simulation models of control systems stabilizing the luminous flux value during the stabilization of thermal conditions occurring after the luminaire switch-on. The determined models were used to develop simulation models of control systems stabilizing the luminous flux value during the stabilization of thermal conditions occurring after the luminaire switch-on. The simulations have confirmed the correct operation of the control system using the developed short-term luminous flux depreciation model.

"Discussion" provides the results of estimating the potential electricity savings from reducing short-term luminous flux depreciation. Necessary for calculating the electricity savings resulting from short-term luminous flux depreciation reduction was the determination of functions approximating power changes. For this purpose, the Curve Fitting Toolbox included in the MATLAB was used. It was assumed that the measure of fitting accuracy is the value of the root mean square error *R*^2^, which should not be less than 0.95. For all analyzed luminaires, this condition was met.

It seems that the annual electricity savings achieved are small for a single luminaire. They are in the range of 0.08 kWh/year for the L1 luminaire to 0.41 kWh/year for the L3 luminaire. When estimating the potential energy savings achieved by reducing short-term luminous flux depreciation, they should be considered in terms of the number of luminaires produced. Assuming that the luminaire can be produced in a series of 20,000 units and will be operated for 15 years, the calculated energy savings are 24 MWh, 36 MWh, 123 MWh, 54 MWh for the L1, L2, L3 and L4 luminaires, respectively. The largest savings were achieved for luminaire L3 because for this luminaire the difference between the initial power and the power after the thermal conditions stabilized is the largest and is equal to 4.67%. The electricity savings achieved translate into reductions in greenhouse gas emissions and other pollutants into the atmosphere. The largest reduction in greenhouse gas and other pollutant emissions occurred for the L3 luminaire and, for example, for CO_2_ is 87.95 tons.

In conclusion, taking into consideration the uncomplicated structure of the LED luminaire model presented in the article and its simple hardware implementation, that the reduction of the phenomenon of short-term depreciation of luminous flux will bring measurable economic (reduction of electricity consumption) and ecological effects, in the form of reduction of greenhouse gas emissions into the atmosphere. In addition, it should be mentioned that a model of the short-term luminous flux depreciation phenomenon of practically any LED luminaire can be developed using the dynamic object identification method.

## Data Availability

The datasets used and analysed during the current study available from the corresponding author (R.S) on reasonable request.
